# Elevated Serum Leptin, Adiponectin and Leptin to Adiponectin Ratio Is Associated with Chronic Kidney Disease in Asian Adults

**DOI:** 10.1371/journal.pone.0122009

**Published:** 2015-03-20

**Authors:** Cynthia Ciwei Lim, Boon Wee Teo, E. Shyong Tai, Su Chi Lim, Choong Meng Chan, Sunil Sethi, Tien Y. Wong, Charumathi Sabanayagam

**Affiliations:** 1 Department of Renal Medicine, Singapore General Hospital, Singapore, Singapore; 2 Department of Medicine, National University of Singapore, National University Health System, Singapore, Singapore; 3 Diabetes Centre, Khoo Teck Puat Hospital, Singapore, Singapore; 4 Department of Pathology, Yong Loo Lin School of Medicine, National University of Singapore, Singapore, Singapore; 5 Singapore National Eye Center, Singapore, Singapore; 6 Ophthalmology and Visual Sciences Academic Clinical Program, Duke-NUS Graduate Medical School, National University of Singapore, Singapore, Singapore; 7 Singapore Eye Research Institute, Singapore, Singapore; University of Leicester, UNITED KINGDOM

## Abstract

**Background:**

Adiponectin and leptin, two of the key cytokines secreted by adipocytes, have been shown to be associated with cardiovascular disease. However, the association of these adipocytokines with chronic kidney disease (CKD) is not clear. We examined the association of serum adiponectin, leptin levels and leptin to adiponectin ratio (LAR) with CKD in a population-based sample of Asian adults.

**Methods:**

We conducted a case-control study (450 CKD cases and 920 controls matched for age, sex and ethnicity) involving Chinese and Indian adults aged 40–80 years who participated in the Singapore Epidemiology of Eye Diseases Study (2007–2011). CKD was defined as an estimated glomerular filtration rate <60 mL/min/1.73m^2^ from serum creatinine. Serum adiponectin and leptin levels were measured using commercially available ELISA. Odds ratio of CKD associated with elevated adiponectin and leptin levels were estimated using logistic regression models adjusted for age, gender, ethnicity, education, smoking, body mass index, diabetes, blood pressure, total and HDL cholesterol.

**Results:**

CKD cases had higher levels of leptin (mean [SD] 9.7 [11.5] vs.16.9 [20.2] ng/mL, p<0.0001) and adiponectin (10.4 [7.4] vs. 9.2 [4.2], p = 0.001) compared to controls. In multi-variable models, compared to those in the lowest quartile, the OR (95% confidence interval) of CKD among those in the highest quartile were: 6.46 (3.84, 10.88), 1.94 (1.32–2.85) and 2.88 (1.78–4.64) for leptin, adiponectin and LAR. Similar associations were also observed when adiponectin and leptin were analyzed as continuous variables. This positive association of serum adiponectin, leptin and LAR with CKD was consistently present in subgroups of gender, ethnicity, diabetes, hypertension and overweight status (all P-interaction >0.1).

**Conclusions:**

Higher levels of serum adiponectin, leptin and LAR were positively associated with CKD independent of traditional risk factors in this Asian population.

## Introduction

Chronic kidney disease (CKD) is common in Asia, with epidemiological studies reporting prevalences of 6–17% [1]. It is associated with increased mortality and cardiovascular disease (CVD), as well as substantial healthcare cost [2–4]. It is thus important to identify patients at risk of CKD and those with early CKD in order to implement timely prevention and treatment. As obesity and insulin resistance are associated with CKD [5,6], there is increasing interest in the relationship between adipokines secreted by adipose tissue and kidney disease. In-vitro and animal studies have demonstrated that adipokines including leptin and adiponectin may mediate pathological and functional changes in renal parenchyma [7–9]. Higher leptin and lower adiponectin levels were shown to be associated with obesity, dyslipidemia, insulin resistance, hypertension and inflammatory states [10–18], conditions which are involved in the pathogenesis of CKD. Elevated leptin levels have also been consistently shown to be associated with CKD in the general population as well as among diabetic and obese non-diabetic patients [11,19–23].

However, in contrast to the finding of an association between lower adiponectin levels and risk factors of CKD, higher adiponectin levels were found in end-stage renal disease (ESRD) patients requiring dialysis [24,25]. The role of adiponectin in non-dialysis dependent CKD is also inconsistent [13,21,22,26–30], with some studies showing lower levels of adiponectin to be associated with CKD, while others showing higher levels to be associated with CKD or no significant association. In this context, the ratio of leptin to adiponectin (LAR) reflecting compromised adipose tissue function has been shown to be a better predictor of adverse outcomes including CVD and mortality in the general population as well as in ESRD patients than either leptin or adiponectin alone [31–33]. However, no previous study has examined the association of LAR with CKD in the general population. We thus aimed to examine the association of leptin, adiponectin and LAR with CKD in a population-based sample of Asian adults.

## Subjects and Methods

We designed a case-control study nested within two large population-based cross-sectional studies: the Singapore Indian Eye Study (SINDI, 2007–2009, n = 3400) and the Singapore Chinese Eye Study (SCES, 2009–2011, n = 3353). Details of the methodology and population characteristics of SINDI and SCES were published previously [34]. In brief, SINDI and SCES each recruited adults aged 40–80 years identified by age-stratified random sampling from computer-generated random lists of 12,000 ethnic Indian and Chinese residents provided by Singapore’s Ministry of Home Affairs. We combined both studies into a single dataset as both followed similar protocols and were conducted in the same study center (Singapore Eye Research Institute).

Cases and controls were defined by the presence and absence of CKD respectively. Controls were frequency-matched to age (within 5 years age group), sex and ethnicity with cases. As we had difficulty finding controls for Chinese cases within the age and sex strata, we chose more Indian controls. However, to account for the age and ethnic differences between cases and controls, we adjusted for age and ethnicity in the regression models. CKD was defined as estimated glomerular filtration rate (eGFR) <60 ml/min/1.73 m^2^. The eGFR was calculated using the serum creatinine-based CKD-EPI formula validated in multi-ethnic Asians [35,36]. Severity of CKD was defined by eGFR categories: <45, 45–60 and >60. An interviewer-administered questionnaire was used to collect participants’ demographic, lifestyle and medical co-morbidity data. Educational level was categorized into primary and below (≤6 years) and secondary and above (>6 years). Cigarette smoking was categorized into current smokers or non-smokers. Alcohol consumption was categorized into ever-drinkers and non-drinkers. Physical examination included weight, height and clinic blood pressure (BP). Systolic BP and diastolic BP were measured using a digital automatic BP monitor (Dinamap model Pro Series DP110X-RW, 100V2; GE Medical Systems Information Technologies Inc., Milwaukee, Wisconsin, USA) after the participant was seated for at least 5 minutes. BP measurement was repeated after a 5 minute interval. A third measurement was made if the systolic BP differed by more than 10 mmHg or the diastolic BP by more than 5 mmHg. The mean between the two closest readings were then taken as the blood pressure for that individual. Body mass index (BMI) was calculated as weight in kilograms divided by the square of height in meters (kg/m^2^). Hypertension was defined as systolic BP ≥140 mmHg or diastolic ≥90 mmHg or self-reported physician-diagnosed hypertension or use of blood-pressure lowering medication. Diabetes mellitus (DM) was defined as casual plasma glucose ≥11.1mmol/L or self-reported physician-diagnosed diabetes or use of glucose-lowering medication. Cardiovascular disease (CVD) was defined as self-reported myocardial infarction, angina or stroke. Serum leptin and adiponectin were measured from stored serum samples using commercially available ELISA kits. Leptin was measured using direct sandwich technique (Millipore, Missouri, USA). The sensitivity of the assay was 0.135 ng/mL + 2SD. The % CV at concentrations ranging from 2.34 ng/ml to 28.9 ng/ml was below 10% (2.6% to 6.2%). Total adiponectin was measured using sandwich ELISA (BioVendor Laboratorní medicína a.s, Brno, Czech Republic) with lower limit of detection at 0.026 μg/mL. The % CV at concentrations ranging from 8.39 ug/ml to 20.79 ug/ml was below 10% (5.9% to 9.9%). LAR was calculated by dividing leptin (ng/ml) by adiponectin (μg/ml). Other laboratory measurements included random plasma glucose, glycated haemoglobin (HbA1c), serum total and high-density lipoprotein (HDL) cholesterol. All laboratory investigations were conducted at the Singapore General Hospital laboratory which is accredited by the College of American Pathologists.

Participants’ written consents were obtained for the questionnaire, physical examination and laboratory investigations at time of enrolment. This study abided by the Declaration of Helsinki and was approved by the Singapore Eye Research Institute Institutional Review Board.

### Statistical analyses

We compared the characteristics of the study participants by case-control status employing the chi-square test or analysis of variance as appropriate for the variable. Leptin, adiponectin and LAR were analyzed in quartiles and also as continuous variables (per SD increase). Analyses were carried out separately for leptin, adiponectin and LAR. First, we examined the association between leptin levels and CKD using logistic regression models adjusted for 1) age, sex, 2) additionally adjusted for ethnicity, educational level, BMI, systolic BP, diabetes, CVD, current smoking, ever drinker, total cholesterol and HDL cholesterol. Trends across the quartiles of leptin levels were examined using the quartiles as an ordinal variable. To examine the consistency of the association between leptin levels and CKD, we performed subgroup analysis stratified by potential confounders including sex, ethnicity, overweight, diabetes and hypertension status. We examined statistical interaction by stratifying variables by including cross-product interaction terms in the corresponding regression models. We then repeated all the above analyses using 1) adiponectin levels and 2) LAR as the exposure. Finally, we estimated the mean leptin and adiponectin levels by severity of CKD (eGFR categories) using analysis of covariance adjusted for potential confounders. In a supplementary analysis, to account for the effect of medication use, we repeated the multi-variable models additionally adjusting for medication used (anti-diabetic, anti-hypertensive or lipid-lowering medication) for leptin and adiponectin. All statistical analyses were performed using SAS version 9.3 (SAS Institute Inc., Cary, NC, USA).

## Results

**Table 1** compares the characteristics of 450 participants with CKD (200 Chinese and 250 Indians) versus 920 controls (488 Chinese and 432 Indians). Since cases were frequency matched in age to controls, cases were older than controls (68.8 vs. 66 years, p<0.0001). Compared to those without CKD, those with CKD were primary/below educated, non-drinkers, had higher prevalence of diabetes, hypertension, and CVD and also had higher levels of BMI, random plasma glucose, HbA1c, serum adiponectin and leptin levels and lower levels of diastolic BP, total cholesterol, HDL cholesterol, LDL cholesterol and triglycerides. Cases were more likely to be receiving anti-diabetic, anti-hypertensive and lipid-lowering medication compared to controls (all p<0.0001), The lower levels of DBP and cholesterol levels observed in cases may be explained by the higher usage of anti-hypertensive and lipid-lowering medication.

**Table 1:** Characteristics comparing CKD cases and controls.

Characteristics	Cases (n = 450)	Controls (n = 920)	p-value
Age, years	68.8 (8.5)	66.0 (9.5)	<0.0001
Female, %	40.4	42.4	0.5
Ethnicity, Chinese %	44.4	53.0	0.003
Primary/below education, %	37.6	31.6	0.03
Current smoker, %	11.6	13.3	0.4
Ever drinker, %	6.4	11.4	0.004
Diabetes, %	46.9	27.2	<0.0001
Hypertension, %	86.2	72.1	<0.0001
Self-reported CVD, %	26.4	14.4	<0.0001
Plasma glucose, mmol/L	7.8 (4.0)	6.9 (3.1)	<0.0001
HbA1c, %	6.6 (1.3)	6.3 (1.1)	<0.0001
Systolic BP, mm Hg	141.8 (21.2)	141.5 (19.9)	0.8
Diastolic BP, mm Hg	74.7 (9.8)	77.1 (9.4)	<0.0001
Total cholesterol, mmol/L	4.9 (1.1)	5.2 (1.1)	<0.0001
HDL cholesterol, mmol/L	1.1 (0.4)	1.2 (0.4)	<0.0001
LDL Cholesterol, mmol/L	2.9 (0.9)	3.2 (0.9)	<0.0001
Triglycerides, mmol/L	1.8 (0.9)	1.9 (1.0)	<0.0001
BMI, kg/m^2^	25.7 (4.8)	24.5 (4.3)	<0.0001
Adiponectin, μg/ml	10.4 (7.4)	9.2 (4.2)	0.0003
Leptin, ng/ml	16.9 (20.2)	9.7 (11.5)	<0.0001
Leptin:Adiponectin ratio (LAR)	2.0 (2.4)	1.2 (1.5)	<0.0001
Anti-diabetic medication, %	37.1	20.1	<0.0001
Anti-hypertensive medication, %	79.3	47.3	<0.0001
Lipid-lowering medication, %	61.1	38.1	<0.0001

P-value represents difference in characteristics between cases and controls by chi-square or analysis of variance as appropriate.

Participants with CKD had higher levels of leptin (mean [SD] 9.7 [11.5] vs.16.9 [20.2] ng/mL, p<0.0001) and adiponectin compared to those without CKD (10.4 [7.4] vs. 9.2 [4.2], p = 0.001). **Table 2** shows the association between serum leptin levels and CKD. A positive association between higher leptin quartiles and CKD were present in both the age- and sex-adjusted model and the multi-variable model. This positive association persisted when serum leptin was analyzed as a continuous variable. variable model. This positive association persisted when serum leptin was analyzed as a continuous variable.

**Table 2:** Association of serum leptin with CKD.

Leptin levels (ng/mL)	Cases (n = 450)	Controls (n = 920)	Age, sex-adjusted OR (95% CI)	Multivariable adjusted OR (95% CI)*
Quartile 1(0.8–3.1)	71	268	Reference	Reference
Quartile 2 (3.1–6.6)	92	240	1.58 (1.10, 2.27)	1.53 (1.05,2.25)
Quartile 3 (6.6–14.5)	126	228	2.86 (1.99, 4.11)	2.78 (1.84, 4.15)
Quartile 4 (14.5–100)	161	184	6.25 (4.17, 9.39)	6.46 (3.84, 10.88)
P for trend			<0.001	<0.001
Per SD increase (15.3)			1.98 (1.69, 2.31)	2.16 (1.75, 2.66)

* Adjusted for age, sex, ethnicity, primary/below education, diabetes, CVD, BMI, systolic BP, current smoking, ever drinker, total cholesterol, HDL cholesterol.

**Table 3** shows the relationship between serum adiponectin and CKD. When analyzed in quartiles, adiponectin levels did not show a significant association with CKD in the age, sex-adjusted model but the association became significant after additional adjustment for factors including presence of diabetes, CVD, systolic BP, smoking and alcohol intake, and total and HDL cholesterol levels, with a significant p-trend. When analyzed as a continuous variable, serum adiponectin was positively associated with CKD in both the age, sex-adjusted and the multi-variable models.

**Table 3:** Association of serum adiponectin with CKD.

Adiponectin levels (ug/mL)	Cases (n = 450)	Controls (n = 920)	Age, sex-adjusted OR (95% CI)	Multivariable adjusted OR (95% CI)*
Quartile 1(2.7–6.6)	98	238	Reference	Reference
Quartile 2 (6.6–8.6)	115	228	1.14 (0.82, 1.59)	1.41 (0.99, 2.00)
Quartile 3 (8.6–11.1)	113	225	1.10 (0.79, 1.54)	1.47 (1.03, 2.11)
Quartile 4 (11.1–77.7)	124	229	1.12 (0.80, 1.57)	1.94 (1.32, 2.85)
P for trend			0.6	0.001
Per SD increase (5.5 units)			1.19 (1.06, 1.34)	1.52 (1.30, 1.77)

* Adjusted for age, sex, ethnicity, primary/below education, diabetes, CVD, BMI, systolic BP, current smoking, ever drinker, total cholesterol, HDL cholesterol.

Similar to leptin, higher LAR quartiles were positively associated with CKD in both the age- and sex-adjusted model and the multi-variable model; and when LAR was analyzed as a continuous variable (**Table 4**).

**Table 4:** Association of serum leptin/adiponectin ratio (LAR) with CKD.

Leptin/adiponectin ratio	Cases (n = 450)	Controls (n = 920)	Age, sex-adjusted OR (95% CI)	Multivariable adjusted OR (95% CI)*
Quartile 1 (0.02–0.36)	151	193	Reference	Reference
Quartile 2 (0.36–0.80)	118	220	1.60 (1.13–2.26)	1.41 (0.98–2.04)
Quartile 3 (0.80–1.86)	104	240	2.25 (1.58–3.20)	1.86 (1.25–2.76)
Quartile 4 (1.86–14.53)	77	267	3.93 (2.71–5.69)	2.88 (1.78–4.64)
P for trend			<0.001	<0.001
Per SD increase (1.1)			1.63 (1.43–1.86)	1.46 (1.23–1.72)

* Adjusted for age, sex, ethnicity, education, diabetes, CVD, BMI, systolic BP, current smoking, ever drinker, total cholesterol, HDL cholesterol

**Table 5** shows that the positive associations between serum adiponectin, serum leptin, LAR and CKD were consistently present within subgroups stratified by gender, ethnicity, BMI, diabetes and hypertension status (all P-interaction >0.5).

**Table 5:** Association of serum leptin, adiponectin and LAR (per SD increase), with CKD within subgroups.

	Multivariable adjusted OR (95% CI)*		
Subgroups	Leptin per 15.3 units increase	Adiponectin per 5.5 units increase	LAR per 1.1 units increase
Men	3.16 (1.94–5.14)	1.43 (1.19–1.72)	1.45 (1.17–1.80)
Women	2.15 (1.67–2.77)	1.73 (1.34–2.23)	1.48 (1.12–1.96)
Chinese	3.50 (2.03–6.05)	1.67 (1.33–2.10)	1.71 (1.33–2.20)
Indians	2.03 (1.61–2.56)	1.48 (1.19–1.85)	1.24 (0.98–1.56)
BMI <25 kg/m^2^	3.56 (2.00–6.34)	1.53 (1.26–1.86)	1.51 (1.19–1.92)
BMI ≥25 kg/m^2^	2.23 (1.75–2.83)	1.56 (1.19–2.03)	1.39 (1.08–1.79)
Diabetes—No	1.93 (1.49–2.51)	1.47 (1.23–1.76)	1.60 (1.28–2.01)
Diabetes—Yes	2.91 (1.97–4.30)	1.78 (1.30–2.44)	1.35 (1.03–1.77)
Hypertension—No	1.50 (0.93–2.41)	2.05 (1.31–3.23)	1.65 (1.04–2.61)
Hypertension—Yes	2.31 (1.82–2.95)	1.44 (1.22–1.69)	1.39 (1.15–1.67)

* Adjusted for age, sex, ethnicity, primary/below education, diabetes, CVD, BMI, systolic BP, current smoking, ever drinker, total cholesterol, HDL cholesterol (P-interaction >0.1 for all stratifying variables for both leptin and adiponectin).

**Fig 1:**
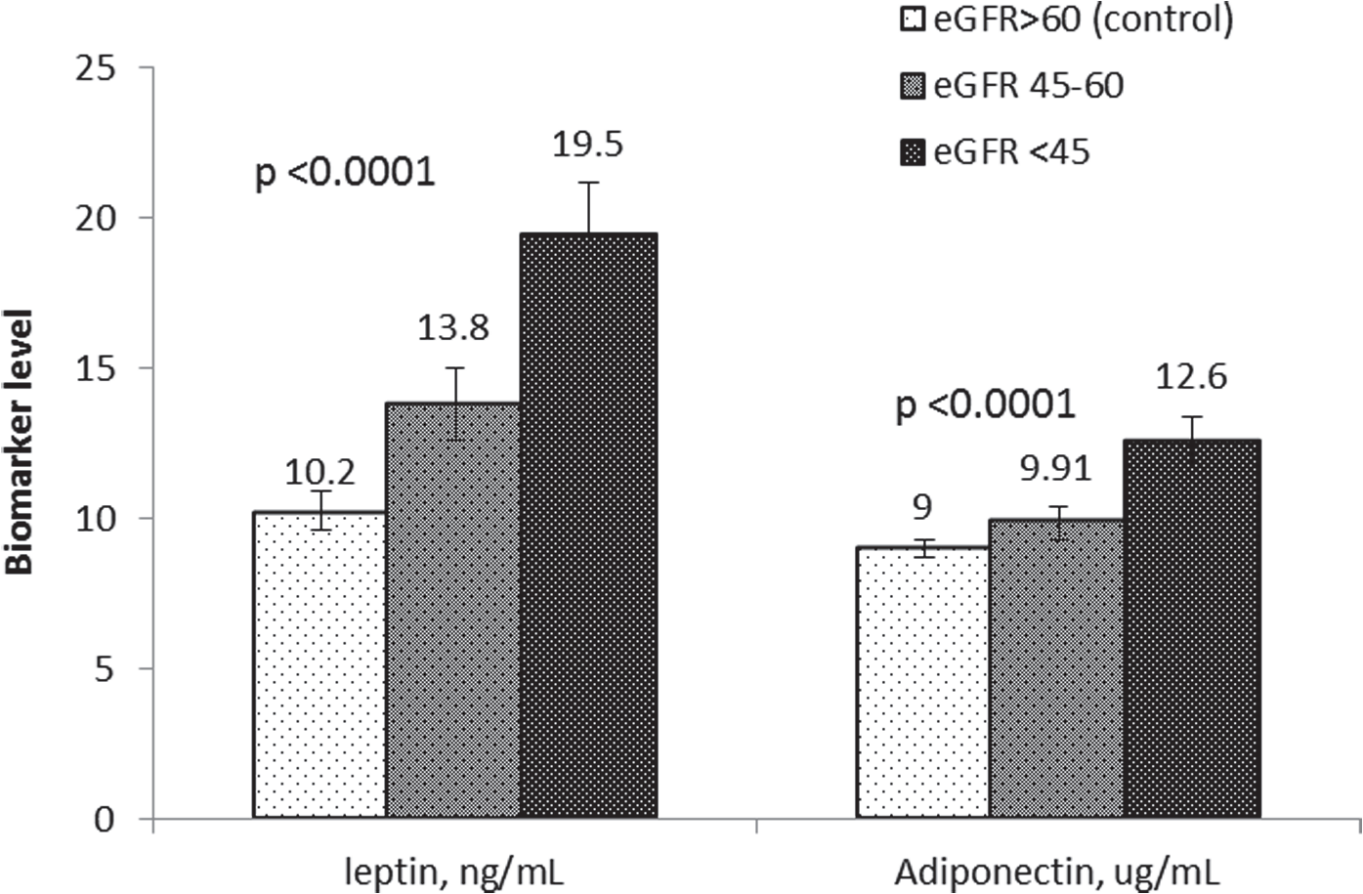
Adjusted* mean leptin and adiponectin levels by severity of CKD defined by eGFR levels. * Adjusted for age, sex, ethnicity, primary/below education, diabetes, CVD, BMI, systolic BP, current smoking, ever drinker, total and HDL cholesterol. p-value represents difference in adjusted mean levels of leptin and adiponectin by eGFR categories.

**Fig. 1** shows the mean levels of leptin and adiponectin by severity of CKD adjusted for the potential confounding factors. Mean levels of both leptin and adiponectin increased significantly in a stepwise fashion across decreasing categories of eGFR (p-trend <0.0001).

In supplementary analyses, when we repeated the multivariable models in Table 2 and 3 additionally adjusting for medication use, the effect estimates were largely similar; compared with quartile 1 of leptin, the multi-variable OR (95% CI) of CKD was 1.36 (0.92–2.02), 2.32 (1.53–3.53) and 5.55 (3.24–9.51) in quartiles 2, 3 and 4; corresponding estimates for adiponectin were, 1.44 (1.01–2.07), 1.55 (1.07–2.25), 1.96 (1.32–2.93) in quartiles 2, 3 and 4.

## Discussion

In this large population-based sample of Asian adults, higher serum leptin, adiponectin levels and LAR were positively associated with CKD, independent of age, gender, education level, BP, diabetes mellitus, CVD, smoking, alcohol intake, and total and HDL cholesterol levels. These associations persisted when leptin and adiponectin were analyzed as continuous variables and remained consistent within subgroups of gender, ethnicity, overweight, diabetes and hypertension status. These findings confirm the previously reported associations of leptin and adiponectin with CKD in Western populations in Asian populations and illustrated the association of LAR with CKD.

Previous studies conducted in predominantly white populations found leptin to be inversely related to GFR [19–21]. In a large study of 5,820 participants from the United States Third National Health and Nutrition Examination Survey (NHANES III), higher plasma leptin concentrations were found to be associated with CKD after adjusting for age, gender, ethnicity, BMI, diabetes, hypertension, and serum cholesterol [20]. As leptin is mainly metabolized by kidney proximal tubular cells, CKD can result in decreased leptin clearance and hence higher circulating leptin levels. Leptin may mediate glomerular hypertrophy and sclerosis by stimulating glomerular endothelial and mesangial cell proliferation and type IV collagen production [7]. Murine studies also found that leptin increased renal sympathetic nerve activity in a dose-dependent manner [37,38]. However, few longitudinal studies are available to prove a causal relationship. A cohort of 804 participants with GFR <55 ml/min/1.73 m^2^ reported that higher leptin levels were not associated with rate of GFR decline [39]. However, the study only included patients with non-diabetic kidney disease and had a relatively short follow up (mean 2.2 years).

Although some earlier studies reported no difference in adiponectin levels between CKD and non-CKD patients, they had small sample sizes [22,30] or did not take into account confounders such as BMI [21,22], age and gender [22]. Obesity is known to be associated with lower adiponectin levels [16,17]. Mills et al compared 201 CKD patients with 201 healthy controls and found that adiponectin levels ≥7.24 μg/ml was not significantly associated with CKD [OR (95% CI), 1.70 (0.98, 2.95), p = 0.06] until after additional adjustment for BMI [2.03 (1.14, 3.61), p = 0.02] [21]. Our results were consistent with other population-based studies that demonstrated higher adiponectin levels in CKD [40,41]. Doumatey et al studied 792 non-diabetic West Africans and found serum adiponectin to be inversely related to GFR (Beta = −0.10) in multivariate regression analysis [40]. Adiponectin was also inversely related to renal function among patients with more severe renal disease not requiring dialysis [42], among patients with diabetes mellitus [26,43,44] and coronary heart disease [29]. In a cohort of 177 patients followed up over 7 years, men with plasma adiponectin concentrations >4μg/ml had faster CKD progression (defined as doubling of baseline serum creatinine and/ or end stage renal failure) [45]. Elevated adiponectin levels may reflect a response to glomerular injury. Sharma et al proposed that adiponectin modulated oxidative stress in glomerular podocytes because adiponectin-deficient mice developed podocyte foot process effacement and albuminuria which improved after adiponectin treatment [9]. Patients with ESRD had increased production of adiponectin with elevated adiponectin mRNA and adiponectin receptor-1 mRNA expression in their adipose tissue [46]. Increased adiponectin levels in CKD may also due to impaired renal clearance since urinary adiponectin levels correlate inversely with GFR and plasma adiponectin levels [30].

LAR was significantly positively associated with CKD in this study, possibly reflecting the greater magnitude of positive association between leptin and CKD compared to adiponectin. Earlier studies have shown LAR to be positively associated with CKD risk factors such as hypertension, obesity and insulin resistance [47–49]. In particular, it appeared to be more predictive of CVD and vascular disease than either leptin or adiponectin alone [32,50,51]. Liao et al evaluated LAR in non-diabetics with cardiac syndrome X, a disease postulated to involve endothelial dysfunction with impaired micro-vascular dilatation and thus shares features with chronic kidney parenchymal disease [32]. Patients with disease had higher leptin levels and LAR than controls after adjusting for BMI and homeostasis model assessment (HOMA) index, suggesting that LAR was a reliable marker of cardiac syndrome X independent of insulin resistance. Similarly, we found that LAR was significantly associated with CKD even after adjustment for history of CVD and markers of insulin resistance such as BMI.

The strengths of our study included a large population-based sample and also took into account and adjusted for many factors that may affect CKD and adipokines levels. To our knowledge, this is the first study to examine the relationship between LAR and renal function. However, our study has some limitations. First, the use of a single measurement of serum creatinine to determine eGFR, without data on albuminuria may have misclassified the status of CKD. Second, we did not assess the inflammatory status of the participants. Inflammation is associated with both leptin and adiponectin and also with CKD. Over-expression of hepicidin induced by interleukin-6 changes the availability of iron and leas to anaemia in CKD patients. It is thus possible that lack of information on inflammatory and hematological markers could have caused residual confounding. Also, the cross-sectional nature of this study means that it cannot determine a causal relationship between adipokines and CKD.

## Conclusions

Serum leptin, adiponectin and LAR were significantly associated with CKD in a large population-based sample of Asian adults, independent of traditional risk factors. Further prospective studies examining the longitudinal relationship as well as studies demonstrating improvement in renal function after lowering adiponectin and leptin levels are required to establish a causal relationship and whether these adipokines can serve as markers of response to treatment.
